# Optimizing chromosome dispersion quality: the key role of cell density

**DOI:** 10.3389/fcell.2025.1636498

**Published:** 2025-07-18

**Authors:** Chao-Xian Gao, Li-Mei Li, Yu-Ting Chen, Ying-Yan Guo, Bo-Xin Li, Xue-Qin Yang, Chang-Ye Hui

**Affiliations:** Pathology and Toxicology Institute, Shenzhen Prevention and Treatment Center for Occupational Diseases, Shenzhen, China

**Keywords:** automated detection, cell density, chromosome dispersion, chromosomal aberrations, radiation detection, homogenization of detection

## Abstract

**Objective:**

This study aims to optimize metaphase dispersion in automated detection by quantitatively determining the optimal cell suspension density to enhance the accuracy and efficiency of chromosomal aberrations analysis.

**Methods:**

Lymphocyte metaphase suspensions were prepared using an automated harvesting system and subjected to a concentration gradient of 10^4^–10^7^ cells/mL. Metaphase images were captured using an automated chromosome scanning and analysis system, and cell density, suspension turbidity, metaphase counts, and dispersion area were measured to quantitatively assess the impact of cell density on metaphase dispersion quality. The practical application of turbidity-based density adjustment was further validated.

**Results:**

The study found that a cell density of 1.04 × 10^6^ cells/mL and suspension turbidity of 0.21 McFarland (McF) yielded the preferred metaphase dispersion, sufficient metaphase counts, and maximum dispersion area, significantly reducing chromosome crossover and overlap. Turbidity adjustment enabled consistent dispersion effects across different initial densities, markedly improving the uniformity of metaphase dispersion.

**Conclusion:**

This study innovatively established a turbidity-based cell density adjustment method, clarifying the impact of cell density on metaphase dispersion through quantitative means and providing standardized technical support for automated detection. This method effectively addresses the inconsistency in metaphase dispersion due to varying cell densities in automated detection, offering a significant basis for homogenizing detection results across laboratories and advancing the standardization and homogenization of chromosomal aberrations analysis techniques.

## 1 Introduction

Radiation workers are chronically exposed to ionizing radiation, and detecting chromosomal aberrations in peripheral blood lymphocytes is a core component in assessing their occupational health risks (Tawn et al., 2018; Kim et al., 2022). Additionally, chromosomal aberrations analysis plays an irreplaceable role in evaluating the condition of casualties, predicting prognosis, and assessing treatment efficacy in nuclear and radiation emergency events (Liu, 2022). However, the accuracy and efficiency of chromosomal aberrations analysis rely heavily on the quality of chromosome dispersion, which is influenced by numerous factors, including temperature and humidity control, as well as cell suspension density (Deng et al., 2003; Kirov et al., 2014).

In recent years, with the continuous advancement of radiation cytogenetics detection technology, chromosomal aberrations detection has transitioned from the traditional manual harvesting and microscopic reading to an “automated + AI reading” mode based on automated metaphase harvesting and automatic microscope scanning platforms (Bi et al., 2019; Shuryak et al., 2022). This shift has significantly enhanced detection efficiency and accuracy while reducing human error. However, automated reading imposes higher demands on metaphase dispersion. Poor dispersion can significantly increase the probability of chromosome crossover and adhesion, leading to more misjudgments (Subramanian et al., 2020). Therefore, optimizing metaphase dispersion is particularly important in automated detection.

In traditional manual slide preparation, environmental factors such as temperature and humidity were key factors affecting metaphase dispersion (Kirov et al., 2014; Yao et al., 2020). However, with the increasing automation of cell harvesting, these environmental factors have become controllable within automated devices. Despite this, cell density, a factor that may significantly impact dispersion quality, is often overlooked. Cell density may affect the dispersion of metaphases and directly impact the number and quality of metaphases scanned. In automated detection, optimizing cell density is crucial for improving metaphase dispersion, thereby enhancing detection efficiency and accuracy.

Currently, there are no reports on the impact of cell density on metaphase dispersion. Therefore, this study aims to investigate the effect of cell density on metaphase dispersion thoroughly, identify the optimal cell density range for achieving good chromosome dispersion morphology, and establish a low-threshold method for adjusting cell density based on suspension turbidity. Ultimately, through a series of quantitative assessments, we aim to significantly improve metaphase dispersion while obtaining sample slides with appropriate dispersion areas and stable numbers of metaphases, thereby promoting standardized slide preparation and adapting to the needs of automated detection.

Since transitioning to automated detection, radiation cytogenetics testing has taken on new vitality. However, with technological upgrades, the accompanying harvesting and slide preparation processes require optimization and improvement. This study was conducted to meet the needs of automated detection, aiming to optimize cell density to enhance metaphase dispersion and provide standardized and reliable technical support for automated reading. Moreover, while our study focuses on optimizing cell density for radiation cytogenetics, the principles and methods we develop broadly apply to other cytogenetic applications, such as prenatal diagnosis (Liu et al., 2022) and leukemia typing (Thakral et al., 2025), where high-quality chromosome dispersion is equally important.

## 2 Materials and methods

### 2.1 Study subjects

Ten healthy volunteers (five males and five females, aged 23–32 years) were selected, all non-radiation workers. Each volunteer provided 2.0 mL of peripheral blood, which was treated with sodium heparin for anticoagulation. This study was approved by the Ethics Committee of the Shenzhen Prevention and Treatment Center for Occupational Diseases (Approval No. LL-202219, Date: 20 May 2022). We obtained written informed consent from all participants before collecting blood samples. Subsequently, 0.5 mL of peripheral blood was inoculated into 3 mL of modified lymphocyte culture medium (Yan et al., 2015), with each blood sample being cultured in four separate tubes. After the completion of the culture, cell suspensions fixed in modified Carnoy’s fixative (ethanol: acetic acid = 3:1) were harvested for subsequent research.

### 2.2 Experimental instruments and reagents

Automated chromosome harvesting system (Chromprep II plus, Shanghai Lechen, China); Automated chromosome dispersion system (Chromprep AS 96, Shanghai Lechen, China); Automated chromosome scanning and analysis system (Zeiss Matafer Axio Imager Z2); Low-speed large-capacity centrifuge (Thermo ST40, United States); Cell counter (Life/COULTER II FL); Cell culture incubator (Thermo 240i, United States); Bacterial turbidimeter (DensiCHEK Plus, bioMérieux). The following reagents were purchased from Sangon Biotech (Shanghai, China): RPMI 1640 culture medium, Gentamicin, colchicine, anhydrous ethanol, glacial acetic acid, and Giemsa stain. Phytohaemagglutinin (PHA) was obtained from Dahuibio (Guangzhou, China).

### 2.3 Experimental methods

#### 2.3.1 Lymphocyte culture and cell suspension preparation

Ten 2 mL peripheral blood samples were collected in our team’s specially designed hourglass-shaped blood collection tube. After incubating at room temperature for 2 h to form a lymphocyte-rich plasma layer, 0.5 mL of the lymphocyte-rich plasma was inoculated into 3.0 mL of modified lymphocyte culture medium containing 2 mM glutamine, 160 mg/mL PHA, and 0.04 mg/mL colchicine, as described previously (Yan et al., 2015). To ensure that the lymphocytes were arrested at the first mitotic division (M-1) for optimal chromosome analysis, colchicine was added to the culture medium at the beginning of the culture. This approach is based on previous studies demonstrating that continuous treatment with colchicine can effectively arrest cells in the first metaphase, thereby providing a pure population of analyzable cells (Chen and Zhang, 1992). Arresting cells at the M-1 phase is crucial for preserving unstable chromosomal aberrations, such as dicentric chromosomes and acentric fragments, indicative of recent DNA damage and essential for accurate cytogenetic analysis.

Each blood sample was inoculated into four tubes, which were then cultured in a tilted centrifuge tube system at 37°C for 48 h. Lymphocyte metaphases were harvested using the automated chromosome harvesting system (Chromprep II plus, Shanghai Lechen, China), yielding 0.5 mL/tube of lymphocyte suspension. The chromosome harvesting procedure was as follows: 3.5 mL of cell culture was centrifuged at 1,200 rpm for 5 min. The supernatant was discarded; 4.5 mL of hypotonic solution (0.075 M potassium chloride) was added, mixed, and treated for 1 min, followed by the addition of 1.5 mL of modified Carnoy’s fixative (ethanol: acetic acid = 3:1), which was then pre-fixed for 1 min. After centrifugation at 1,200 rpm for 5 min and discarding the supernatant, 5 mL of fixative was added, mixed, and immediately centrifuged at 1,200 rpm for 3 min. The supernatant was discarded, and the fixation step was repeated three times, with the final step retaining 0.5 mL of fixative in the centrifuge tube.

#### 2.3.2 Establishment of cell density gradient

After inoculation, culture, and harvest of each whole blood sample, 40 cell suspensions were obtained from the 10 samples. One suspension from each sample was taken and stored at 4°C for subsequent validation experiments. The remaining 30 suspensions were pooled into a 15 mL centrifuge tube to create a series of density gradient suspensions. Specifically, the pooled suspensions were centrifuged at 2,000 rpm for 5 min, with excess supernatant fixative removed to retain only 2 mL of high-density cell suspension. This suspension was then mixed thoroughly. Eight glass bacterial turbidity tubes were taken and numbered 1–8. Tube 1 received 2 mL of the original concentrated cell suspension, while tubes 2–8 each received 1 mL of fixative. 1 mL of the concentrated cell suspension from tube 1 was transferred to tube 2 and mixed thoroughly. Then, 1 mL of the diluted cell suspension from tube 2 was transferred to tube 3, and this process was repeated until tube 8, resulting in a series of 2-fold diluted cell suspensions.

#### 2.3.3 Measurement of suspension turbidity and cell density

After mixing the cell suspensions, the turbidity of the suspensions in tubes 1–8 was measured using a bacterial turbidimeter (DensiCHEK Plus, bioMérieux), with turbidity expressed in McFarland (McF) standards. Simultaneously, 10 µL of cell suspension was drawn with a pipette and added to a cell counting chamber. The cell density of tubes 1–8 was measured sequentially using a cell counter (Life/COULTER II FL), with cell density expressed in cells/mL.

#### 2.3.4 Chromosome image acquisition

The cell suspensions from tubes 1–8 were used to prepare slides with an automated chromosome dispersion system (Chromprep AS 96, Shanghai Lechen, China) under the same temperature and humidity conditions, ensuring uniform dispersion of lymphocyte metaphases across the entire slide. Two sample slides were prepared for each sample. The automated chromosome dispersion system parameters were set as follows: temperature 32°C, humidity 60%, and a drop volume of 50 µL of cell suspension. The prepared slides were air-dried and aged. The slides were then stained with 10% Giemsa stain for 15 min at 25°C, rinsed with running water, and air-dried. The automated chromosome scanning and analysis system (Zeiss Matafer Axio Imager Z2) was used to scan the entire slide at 100× magnification, capturing low-magnification metaphase images and counting the number of metaphases on the entire slide. From the center of each slide, 50 chromosome metaphase images were sequentially selected, preferably those displaying circular or elliptical shapes. If fewer than 50 metaphases were available on the entire slide, all qualified metaphases were selected, and high-magnification (630×) images of the selected metaphases were captured.

#### 2.3.5 Evaluation of metaphase dispersion quality

Dispersion area measurement: Using ImageJ software (National Institutes of Health, United States), the dispersion area of chromosome metaphases was measured by selecting the metaphases with an elliptical selection tool and measuring the size of the selected area.

Chromosome crossover counting: Based on high-magnification images of metaphases, two individuals manually recorded and verified the number of crossover points in each chromosome metaphase.

### 2.4 Determination of optimal cell suspension density

Based on the number of metaphases obtained on the entire slide and the evaluation of metaphase dispersion quality, the cell density with the largest metaphase dispersion area and the fewest chromosome crossovers and adhesions was chosen as the preferred cell density for slide preparation and used for subsequent validation experiments.

### 2.5 Application of optimal density method in chromosome dispersion

The 10 retained cell suspensions used for validation experiments were quantified to 0.5 mL, and their turbidity (t) was measured using a bacterial turbidimeter. If the turbidity (s) of the optimally dispersed cell suspension was known, the volume of fixative to be added was calculated using formula *K* = 0.5 × (*t*/*s*−1). A pipette was then used to add *K* mL of freshly prepared fixative to dilute the cell suspension to the target concentration; if *K* was negative, the corresponding volume of fixative was discarded to concentrate the cell suspension to the target concentration. Chromosome high-magnification images were obtained using the method described in Section 2.3.4, and the dispersion quality of chromosomes was evaluated using the method described in Section 2.3.5.

### 2.6 Statistical analysis

Fifty chromosome metaphases were photographed for each slide. The chromosome dispersion area data followed a normal distribution and were expressed as mean ± standard deviation, with intergroup comparisons made using one-way analysis of variance. The chromosome crossover and overlap data, being count data, were expressed as median (quartiles) and analyzed using non-parametric tests.

## 3 Results

### 3.1 Relationship between cell density and number of metaphases

**FIGURE 1:**
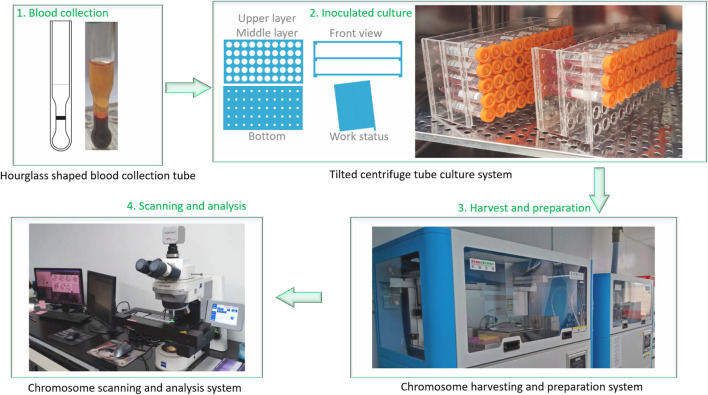
The chromosomal aberrations detection process in our laboratory. 1. Blood collection using a proprietary “hourglass-shaped” blood collection tube (Chinese Utility Model Patent, No. ZL201320496469.X), which separates to obtain a plasma layer rich in peripheral blood mononuclear cells (PBMCs); 2. Inoculation of 0.5 mL of PBMC-rich plasma into the culture medium using a proprietary tilted centrifuge tube culture system (Chinese Utility Model Patent, No. ZL201520203672.2) for cultivation; 3. Automated harvest, slide preparation, and staining under controlled conditions; 4. Analysis of chromosome metaphases using an automated analysis system.

The detailed process of chromosomal aberrations detection in our laboratory, including blood collection, culture, harvest, slide preparation, and scanning analysis, is shown in Figure 1.

**FIGURE 2:**
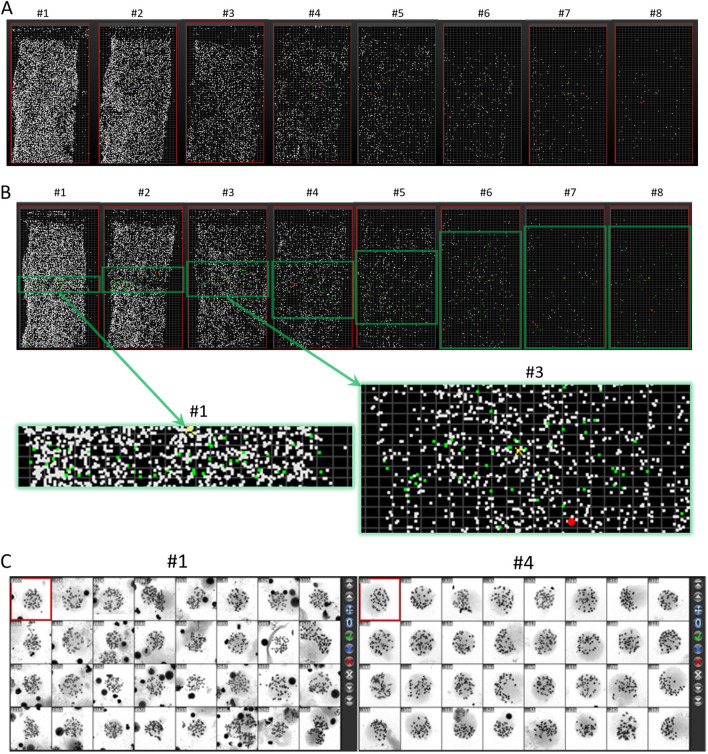
Metaphase maps of slides prepared with serially diluted cell suspensions. **(A)** Moreover, **(B)** shows the scanning results of two slides prepared with the same density gradient of cell suspensions at 100× magnification. Slides 1-8 represent the serially diluted suspensions from left to right. The green frame indicates the selected area for imaging metaphases at 630× magnification, with enlarged views of the imaging areas from slides #1 and #3, where green dots represent the distribution of selected metaphases. **(C)** Shows the metaphase images taken at #1 and #4 dilution levels.

When scanning the entire slide at 100× magnification, the metaphase map obtained is shown in Figure 2. It can be seen that the overall dispersion of the two slides prepared at each cell density is consistent. As shown in Figure 2A, from left to right, with the dilution of the cell suspension, the density of metaphases on the chromosome slide gradually decreases. We selected one group to evaluate dispersion effects by taking images of metaphases at 630× magnification. The imaging area is shown in Figure 2B, focusing on the central square area. As the cell density decreases, the imaging area gradually expands across the entire slide. As shown in Figure 2C, the dispersion morphology of metaphases is closely related to the density of the cell suspension. For example, in the slides prepared with the #1 tube suspension, the high density of metaphases and small dispersion area often results in two or more metaphases being captured in a single image. In contrast, in the slides prepared with the #4 tube suspension, the larger dispersion area means that most captured images contain only one metaphase.

### 3.2 Quantitative analysis of chromosome dispersion effects

**FIGURE 3:**
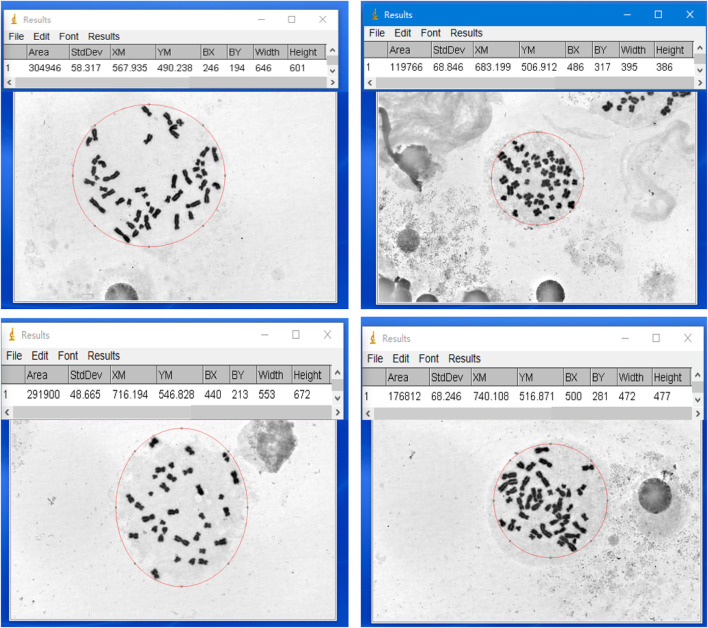
Examples of measuring the dispersion area of metaphases using ImageJ software. Four representative metaphases are shown, with the measured chromosome dispersion area indicated above each example in pixel area (pix).

We established a quantitative method for assessing the dispersion area of metaphases using ImageJ software. Specifically, we opened the high-magnification images of metaphases in ImageJ, selected the metaphases with an elliptical selection tool, adjusted the selection frame to enclose all visible chromosomes within the smallest possible ellipse, and measured the size of the selected area using the measurement tool in the analysis menu (see Figure 3).

**FIGURE 4:**
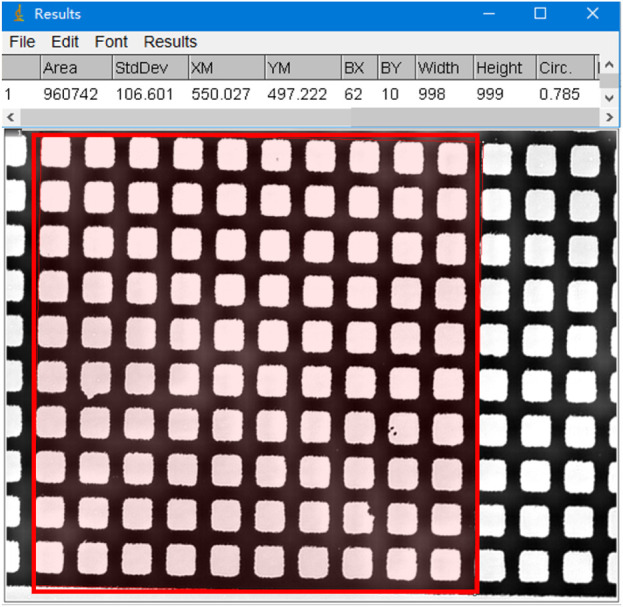
An example of measuring the pixel area of a region containing 100 squares (10 µm × 10 µm) using ImageJ software (red frame shown).

The area measured by ImageJ is the pixel area of the ellipse. To convert this to actual area, as shown in Figure 4, we used a microscope eyepiece micrometer to measure the pixel area of 100 squares (10 μm × 10 μm, with an area of 10,000 μm^2^), which was found to be 960,742 pix. Thus, the conversion relationship between pixel area and actual area is 1 μm^2^ = 96.0742 pix. For example, if the measured pixel area is 153,776 pix, the actual area is 153,776/96.0742 = 1,601 μm^2^.

**FIGURE 5:**
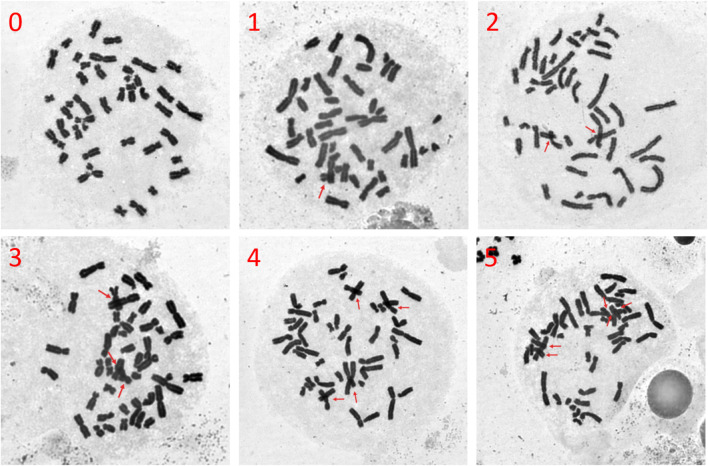
Representative images of chromosome crossover due to poor dispersion of metaphases (magnified 630×). Red arrows indicate chromosome crossover, overlap, and superposition, with the number in the upper left corner representing the count of these occurrences in the metaphase.

Chromosome crossover and overlap are important causes of false positives in chromosomal aberrations identification. Therefore, we imaged metaphases at 630× magnification for each dilution and manually counted the number of chromosome crossovers in each of the 50 selected metaphases. Representative images of chromosome crossovers are shown in Figure 5, and we performed counting to enable quantitative comparison.

### 3.3 Comprehensive evaluation of the effect of cell density on chromosome dispersion

We quantified several key indicators to systematically investigate the impact of cell suspension density on slide preparation quality. Table 1 summarizes the quantitative analysis of the effect of cell suspension density on chromosome dispersion.

**TABLE 1:** Quantitative analysis of the effect of cell suspension density on chromosome dispersion.

Tubes	Cell suspension proportion (%)	Cell density (cells/mL)	Suspension turbidity (McF)	Metaphase dispersion area (μm^2^)	Metaphase number (per slide)	Crossover and overlap count (per metaphase)
#1	100	2.10 × 10^7^	3.74	1,445 ± 331^a^	13,708	1 [1,3]^e^
#2	50	1.04 × 10^7^	1.89	1,710 ± 410^b^	9,587	2 [0,3]^e^
#3	25	3.49 × 10^6^	0.97	2,088 ± 462^c^	4,771	1 [0,2]^e^
#4	12.5	1.92 × 10^6^	0.45	2,232 ± 486^c^	2,622	0 [0,1]
#5	6.23	1.04 × 10^6^	0.21	2,558 ± 510^d^	1,286	0 [0,1]
#6	3.125	4.52 × 10^5^	0.10	2,425 ± 646^d^	509	0 [0,1]
#7	1.5625	2.87 × 10^5^	0.06	2,101 ± 475^c^	235	0 [0,1]
#8	0.78125	9.97 × 10^4^	0.02	1,985 ± 468^c^	122	1 [0,1]^e^

Notes: a, b, c, and d indicate no significant differences within the same group according to one-way ANOVA, but there are significant differences between different groups; e indicates significant differences compared to the sample in tube #5. The chromosome crossover and overlap counts, which follow a non-normal distribution, are represented by quartiles, i.e., median [25%, 75%].

**FIGURE 6:**
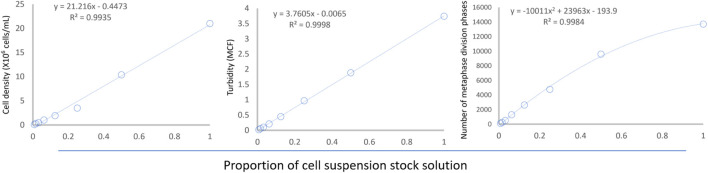
Relationship between cell suspension dilution and cell density, suspension turbidity, and number of metaphases. The regression equations are shown in the upper left corner of the figure.

As the proportion of the original cell suspension decreased stepwise (from tube #1 to tube #8), cell density, suspension turbidity, and the number of scanned metaphases showed a downward trend. Cell density and suspension turbidity exhibited a linear relationship with dilution (see Figure 6). Interestingly, the number of metaphases captured by the automated reading system showed a nonlinear relationship with dilution.

**FIGURE 7:**
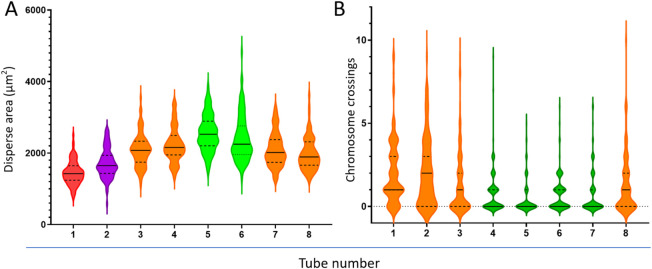
Relationship between cell suspension dilution, metaphase dispersion area **(A)**, and chromosome crossover count **(B)**. Different colors represent significant differences between groups.

Notably, the dispersion area of metaphases increased initially and then decreased with the reduction of cell density in the drop (Figure 7A). The dispersion area reached a maximum of 2,558 ± 510 μm^2^ at a cell density of 1.04 × 10^6^/mL (tube #5). However, further dilution of the cell suspension (tubes #6-#8) decreased the dispersion area. Additionally, the number of chromosome crossovers and overlaps in metaphases was negatively correlated with the dispersion area (Figure 7B), meaning that fewer crossovers and overlaps occurred in metaphases with larger dispersion areas (tubes #4-#7).

For occupational health checks of radiation workers, there is generally required to be more than 500 metaphases on the entire slide for preliminary screening to ensure that at least 100 metaphases with satisfactory morphology are available for analysis (Lopes et al., 2025). Under controllable slide preparation conditions of temperature and humidity, we comprehensively quantified the effect of cell suspension density on dispersion area. We identified that tube #5, with a cell density of 1.04 × 10^6^/mL and corresponding suspension turbidity of 0.21 McF, yielded slides with a balanced number of metaphases and dispersion area. We subsequently conducted validation experiments based on this condition.

### 3.4 Validation experiment

To verify the practical effectiveness of the determined optimal suspension turbidity (0.21 McF), we selected 10 cell suspensions with original turbidity ranging from 0.27 to 1.14 McF for validation experiments. Using formula *K* = 0.5 × (*t*/0.21–1), we calculated the volume of fixative to be added to adjust the suspension turbidity to the target value of 0.21 McF. We then compared the number of metaphases captured and the dispersion area in the slides prepared before and after adjustment (Table 2).

**TABLE 2:** Validation results of the optimal density for chromosome dispersion.

Samples	Original turbidity (McF)	Metaphase number (per slide)	Original dispersion area (μm^2^)	Fixative added (mL)	Adjusted metaphase number (per slide)	Adjusted dispersion area (μm^2^)
1	0.27	709	2,511 ± 992	0.14	661	2,533 ± 696
2	0.36	917	2,279 ± 819	0.36	601	2,595 ± 840
3	0.9	2,269	2,036 ± 455	1.64	636	2,478 ± 714
4	1.14	2,926	1,930 ± 520	2.21	672	2,494 ± 880
5	0.89	2,197	2,014 ± 770	1.62	600	2,414 ± 982
6	0.72	1,784	2,107 ± 740	1.21	643	2,517 ± 715
7	1.13	2,907	2,080 ± 749	2.19	674	2,413 ± 976
8	1.08	2,730	2,079 ± 817	2.07	670	2,503 ± 831
9	0.74	1,855	2,121 ± 649	1.21	693	2,382 ± 581
10	0.45	1,176	2,448 ± 668	0.57	619	2,459 ± 642

Results showed that differences in suspension turbidity led to significant heterogeneity in the dispersion area of metaphases. As suspension turbidity increased, the number of captured metaphases rose, but the dispersion area of metaphases decreased, which is not conducive to subsequent chromosome analysis. Although some samples had small and uneven dispersion areas due to high cell density before adjustment, after adjusting the suspension turbidity to 0.21 McF, the number of metaphases captured on the slides became stable and appropriate, the dispersion area significantly increased, and most importantly, the consistency between samples was significantly improved.

## 4 Discussion

To meet the demand for the number of metaphases required for chromosomal aberrations analysis, our laboratory previously established an improved culture system based on tilted centrifuge tube cultivation and successfully implemented a “one-tube” preprocessing mode from blood inoculation, cell culture, harvest to final slide preparation (Yan et al., 2015). In this system, only 0.5 mL of blood sample is inoculated into 3–4 mL of modified culture medium. Even with a smaller inoculation and culture volume than traditional methods, obtaining an adequate number of lymphocyte metaphases for further analysis is still possible. This is possible by avoiding interference from external animal serum and enhancing aeration and nutrient exchange through tilted cultivation. However, with the continuous development of “automated + AI reading” technology, although the cell harvesting process has been standardized and controlled, there are still significant differences in the number and dispersion quality of metaphases in samples from different subjects after culture and slide preparation, which poses a challenge to the homogenization of test results among different laboratories (Livingston et al., 2006; Bi et al., 2019).

The preparation of chromosomal aberrations analysis slides is a complex process influenced by multiple factors, and each step can affect the final slide quality (Hliscs et al., 1997; Ami et al., 2014; Chomik et al., 2016). However, even under the automated harvesting system where the two key factors affecting slide preparation, temperature, and humidity, are controllable, there are still significant differences in the dispersion of metaphases (Martin et al., 2007). The drop process directly determines the dispersion of chromosomes in metaphase on the slide. Secondary factors affecting chromosome dispersion, such as slide cleanliness, drop height, slide temperature, the amount of water in the wet slide method, and the tilt angle of the slide, can all be effectively controlled in an automated disperser (Dai et al., 2018). At this point, the density of the lymphocyte suspension obtained due to individual differences among subjects becomes the only variable affecting dispersion. However, no studies have focused on the impact of the density of cells and other formed components in the suspension on the dispersion of metaphases. Therefore, we conducted an in-depth investigation of the single variable of cell suspension density.

To systematically investigate the effect of cell suspension density on chromosome dispersion, we quantified all the indicators under study. To ensure the uniformity of the suspension dilution gradient, we used a two-fold dilution method (Hui et al., 2021) and successfully directly counted the lymphocytes fixed with Carnoy’s fixative in the suspension using a cell counter. However, considering that the detection of the cell counter requires special experimental consumables and the detection process is relatively complex, we also examined the bacterial turbidimeter based on the light scattering principle. It can assess the turbidity of the suspension by measuring the intensity of light scattering by the suspension (Wang et al., 2024), which is expected to reflect the density of fixed lymphocytes in the suspension indirectly. As we expected, the results in Figure 6 confirmed that both the lymphocyte count measured by the cell counter and the suspension turbidity measured by the bacterial turbidimeter showed a good linear regression relationship with the dilution degree. This quantitative detection method for cell density laid the methodological foundation for our subsequent adjustment of cell density.

Based on the whole slide scanning results (Figure 2C), we found that when the cell density of the suspension used for dropping is too high, two or more metaphases that are too close may be mistakenly regarded as a single entity by the automated scanning system. Therefore, the linear relationship between the number of identified metaphases and cell dilution degree is poor, especially when the cell suspension density is high, however, the number of captured metaphases is low (Figure 6). However, using a quadratic polynomial, the number of metaphases can be regressed with the dilution degree. Up to this point, we have also evaluated the relationship between cell density and the number of captured metaphases quantitatively. Considering that chromosome crossover, overlap, and adhesion are important factors affecting aberration detection (Lusiyanti et al., 2019), we further established a quantitative evaluation method for chromosome crossover based on the metaphases captured at high magnification (Figure 5) for subsequent dispersion effect evaluation.

To establish a quantitative evaluation method for metaphase dispersion area, we used ImageJ software, a free morphological detection tool (Palomar et al., 2025), to outline complete metaphases with an ellipse, and ImageJ directly provided the pixel area of the ellipse, which was then converted to the actual area value based on the formula we established. Based on this innovative quantitative evaluation method for dispersion area, we were pleasantly surprised to find that as the cell suspension was diluted, the dispersion area of metaphases did not simply increase but showed an approximate “parabolic” trend (Figure 7A), which further highlighted the importance of adjusting cell suspension density for slide preparation effects. Significantly, the number of chromosome crossovers was negatively correlated with the dispersion area of metaphases (Figure 7B). In subsequent investigations, while ensuring that sufficient metaphases were scanned for analysis, we focused on the dispersion area and ultimately determined the preferred cell suspension turbidity value of 0.21 McF and conducted application validation (Table 2). Despite significant differences in metaphase dispersion caused by different initial cell concentrations, stable dispersion effects could be achieved by adjusting a single factor, the cell suspension density.

In traditional manual preparation of lymphocyte metaphases, the volume of fixative retained before dropping is usually fixed (Deng et al., 2003). Even by visually observing the amount of precipitated cells to adjust the volume of fixative, the precision is still limited and more difficult to standardize and promote. In this study, we established a simple quantitative evaluation method for cell density in suspension based on a turbidimeter, which can be easily integrated into an automated disperser. By introducing a turbidity photoelectric detection module for the dropping suspension and coupling it with the adjustment of fixative volume, the uniformity of cell density in the dropping suspension can be achieved, thereby eliminating individual differences among subjects and potentially realizing the homogenization of tests among different laboratories. The innovative quantitative evaluation methods we established based on ImageJ software for dispersion area detection and high-magnification images for chromosome crossover evaluation provide important references for subsequent genetic testing.

## 5 Conclusion

This study systematically analyzed the relationships between cell density, suspension turbidity, number of metaphases, and dispersion area. The results showed that a cell density of 1.04 × 10^6^/mL and a suspension turbidity of 0.21 McF yield optimal chromosome dispersion, with sufficient metaphase counts and minimal chromosome crossover and overlap. The turbidity adjustment method effectively standardizes cell suspension density, enhancing the consistency of chromosome dispersion. The proposed method is simple to operate and well-suited for automated chromosome dispersion and reading systems, providing reliable support for automated cytogenetic detection. It is anticipated that the accuracy and efficiency of chromosomal aberrations analysis will improve, fostering the standardization of radiation cytogenetic detection technology. This method applies to cytogenetic analyses, such as prenatal diagnosis and leukemia typing, where high-quality chromosome dispersion is essential. By enhancing chromosome dispersion quality, our method can contribute to more accurate and efficient genetic diagnostics across various applications.

## Data Availability

The original contributions presented in the study are included in the article/supplementary material, further inquiries can be directed to the corresponding author.
